# Supplementary material for: Increased sensitivity of malaria parasites to common antimalaria drugs after the introduction of artemether-lumefantrine: Implication of policy change and implementation of more effective drugs in fight against malaria
Source: PLoS One. 2024 Jun 20;19(6):e0298585. doi: 10.1371/journal.pone.0298585 (PMC11189199; doi:10.1371/journal.pone.0298585)
Supplement: Attachment — Submitted filename: Response to Reviewers Rebuttal Letter_20231127.docx [file pone.0298585.s007.docx]

Temporal trends of Plasmodium falciparum multi-drug resistance protein 1 gene mutations during implementation of artemisinin combination therapies in Kenya between 2008 and 2019

We've checked your submission and before we can proceed, we need you to address the following issues:

1. We note that the grant information you provided in the ‘Funding Information’ and ‘Financial Disclosure’ sections do not match.

Response: concur. This has been rectified

When you resubmit, please ensure that you provide the correct grant numbers for the awards you received for your study in the ‘Funding Information’ section.
Response: concur. This has been rectified

2. Please ensure that you refer to Table 4 in your text as, if accepted, production will need this reference to link the reader to the Table.

Response: Concur. Sentence 294 has since been rephrased to read, “… respectively, implying increasing parasite sensitivity to the drugs over time (Table 3, Table 4 and …”

Please submit your revised manuscript by Nov 10 2023 11:59PM.

Please include the following items when submitting your revised manuscript:

•	A rebuttal letter that responds to each point raised by the academic editor and reviewer(s). You should upload this letter as a separate file labeled 'Response to Reviewers'.

•	A marked-up copy of your manuscript that highlights changes made to the original version. You should upload this as a separate file labeled 'Revised Manuscript with Track Changes'.

•	An unmarked version of your revised paper without tracked changes. You should upload this as a separate file labeled 'Manuscript'.

When submitting your revision, we need you to address these additional requirements.

1.	Please ensure that your manuscript meets PLOS ONE's style requirements, including those for file naming. The PLOS ONE style templates can be found at

https://journals.plos.org/plosone/s/file?id=wjVg/PLOSOne_formatting_sample_main_body.pdf and

https://journals.plos.org/plosone/s/file?id=ba62/PLOSOne_formatting_sample_title_authors_affiliations.pdf

2.	We note that the grant information you provided in the ‘Funding Information’ and ‘Financial Disclosure’ sections do not match. When you resubmit, please ensure that you provide the correct grant numbers for the awards you received for your study in the ‘Funding Information’ section.

3.	Thank you for stating the following financial disclosure:

[Funding for this study was provided by the Armed Forces Health Surveillance Branch (AFHSB) and its Global Emerging Infections Surveillance (GEIS) Section, Grant P0117_22_KY. The study sponsors had no role in study design; in the collection, analysis, and interpretation of data; in the writing of the report; and in the decision to submit the paper for publication. The corresponding author should confirm that he or she had full access to all the data in the study and had final responsibility for the decision to submit for publication.]

Please state what role the funders took in the study. If the funders had no role, please state: ""The funders had no role in study design, data collection and analysis, decision to publish, or preparation of the manuscript.""

If this statement is not correct you must amend it as needed.

Please include this amended Role of Funder statement in your cover letter; we will change the online submission form on your behalf

Response: Added in the cover letter

4.	In your Data Availability statement, you have not specified where the minimal data set underlying the results described in your manuscript can be found. PLOS defines a study's minimal data set as the underlying data used to reach the conclusions drawn in the manuscript and any additional data required to replicate the reported study findings in their entirety. All PLOS journals require that the minimal data set be made fully available. For more information about our data policy, please see http://journals.plos.org/plosone/s/data-availability.

""Upon re-submitting your revised manuscript, please upload your study’s minimal underlying data set as either Supporting Information files or to a stable, public repository and include the relevant URLs, DOIs, or accession numbers within your revised cover letter. For a list of acceptable repositories, please see http://journals.plos.org/plosone/s/data-availability#loc-recommended-repositories. Any potentially identifying patient information must be fully anonymized.

Response: Thank you for the observation. The minimal data set underlying the results described in our manuscript can be found as Supporting Information files (S3-6).

Important: If there are ethical or legal restrictions to sharing your data publicly, please explain these restrictions in detail. Please see our guidelines for more information on what we consider unacceptable restrictions to publicly sharing data: http://journals.plos.org/plosone/s/data-availability#loc-unacceptable-data-access-restrictions. Note that it is not acceptable for the authors to be the sole named individuals responsible for ensuring data access.

We will update your Data Availability statement to reflect the information you provide in your cover letter.

Response: well noted. No ethical no legal restrictions

5.	Your ethics statement should only appear in the Methods section of your manuscript. If your ethics statement is written in any section besides the Methods, please move it to the Methods section and delete it from any other section. Please ensure that your ethics statement is included in your manuscript, as the ethics statement entered into the online submission form will not be published alongside your manuscript.

Response: Thank you for the comment. This observation is well noted. Our ethics statement is included in the manuscript methods section under the first paragraph of study sites and sample collection section page 6 line 214-217 and has also been deleted after the acknowledgement section.

6.	Please review your reference list to ensure that it is complete and correct. If you have cited papers that have been retracted, please include the rationale for doing so in the manuscript text, or remove these references and replace them with relevant current references. Any changes to the reference list should be mentioned in the rebuttal letter that accompanies your revised manuscript. If you need to cite a retracted article, indicate the article’s retracted status in the References list and also include a citation and full reference for the retraction notice.

Response: Reference list checked one by one to ensure it was complete and correct.

List also updated from 27 to 59. References 28, 34 and 53 added.

Additional Editor Comments:

The authors should additionally, address the following questions.

7.	Artemether and Lumefantrine, are they active antimalarial compounds or their metabolites?

Thank you for the comment. Both artemether and lumefantrine are not the active metabolites but were tested as such. The active metabolite of Artemether is dihydroartemisinin while that of lumefantrine is desbutyl-lumefantrine. A study by Salman and coworkers show that a combined desbutyl-lumefantrine and lumefantrine AUC0–∞ weighted on in vitro antimalarial activity was inversely associated with recurrent parasitemia, suggesting that both the parent drug and the metabolite contribute to the treatment outcome of artemether-lumefantrine. (Salman et al 2011).

ii) please justify their use in in vitro assays in this study

Thank you for the comment. Resistance to antimalarial drugs including lumefantrine in Southeast Asia (SEA), Oceania and Africa have been associated with polymorphisms in Pfmrp1 codons; 191, 437, 876, 1390. The mutations at the 4 codons are already established and validated non-synonymous SNPs frequent in the populations and are associated with resistance to the chosen drugs. Evidence of positive selection on the Pfmrp1 gene and its association with reduced sensitivities of parasites to the selected antimalarials was based on the following studies (Dahlström et al., 2009; Gupta et al., 2014b; Pirahmadi, Zakeri, Afsharpad, & Djadid, 2013; Veiga et al., 2011; Zhao et al., 2019). However, in Kenya this genotypic and the impact on parasite susceptibility to selected antimalarials is scanty. Therefore, this study was conducted to fill this gap to inform policies on malaria prevention interventions. These molecules were assayed for in vitro because other studies have previously reported their in vitro readouts in different transmission from those for this study. Similarly, this study intended to measure the response of isolated from natural infections occurring in the study area so as to provide parallel results.

8.	Why is CQ IC50 lower than that of QN? provide justification and how quality control was ensured during the assays

Response: Thank you for the comment. This observation in our parasite population is due to the withdrawal of CQ drug pressure from the parasites at the onset of sulfadoxine-pyrimethamine (SP) use in Kenya (Balikagala et al., 2020) and increased use of AL (Mwai et al., 2009; Balikagala et al., 2020) which has further selected for parasites that are susceptible to chloroquine. However, QN is still used to date in the treatment of severe malaria. The quality control IC50s threshold of QN is also a bit higher in QN as compared to CQ. Reference standard for sensitivity was carried out by testing of reference strains with well characterized profiles for example Dd2 (Mefloquine sensitive), D6, 3D7 clone is sensitive to chloroquine drug with standard cut off of <10ng/ml and < 45ng/ml in field isolates while W2 is resistant to chloroquine with cut off value of >45ng/ml. Mefloquine cut off in field isolates < 10ng/ml and in W2 clone. Quinine cut off is <275ng/ml. Also the starting concentrations in the drug testing for quinine is high, the concentration ranges (ng/mL), CQ (1,000 to 1.953), and QN (2,000 to 3.906), because chloroquine is more potent than quinine. This finding are in line with what has been reported previously (Zhao et al., 2022).

9.	Provide more information on the implications of the findings of this study on use of Artemisinin based therapies (ACTs) and CQ for malaria treatment.

a. Response: Thank you for the comment. Our study findings show that malaria parasites among Kenyan populations are still sensitive to AQ, QN, CQ, ART, LU and MQ. Despite the presence of Pfmrp1 mutations in parasites among the population. Based on the selected antimalarials comprising ACTs, they have proven to be still effective for malaria treatment in Kenya. CQ can also be possibly reused in future since its sensitivity has improved over time following the release of drug pressure on the parasites.

Review Comments to the Author

Please use the space provided to explain your answers to the questions above. You may also include additional comments for the author, including concerns about dual publication, research ethics, or publication ethics. (Please upload your review as an attachment if it exceeds 20,000 characters)

Reviewer #1: The manuscript tackles a good topic in Antimalarial drug resistance and its relevant because it looks at a gene that is rarely explored. The authors did the standard experiments for checking for in vitro and ex vivo sensitivities. I had a few concerns when it came to these assays

1.	In vitro and ex vivo assays were not differentiated in the manuscript. As we know, during culture adaptation, some clones (in my experience most Lumefantrine tolerant strains), tend to be lost during culture, so these samples should be separated in the analysis.

Thank you for the comment. Our analysis did not separate ex vivo and in vitro samples because in previous studies isolates subjected to ex vivo and in vitro assays have shown marginal difference IC50 (Akala et al., 2011), meaning there is no difference in terms of parasite response to antimalarials in both assays. Based on these findings, all our subsequent studies on drug susceptibility assays have analyzed these samples using the approach adopted in this study (Akala et al., 2011, Eyase et al., 2013, Wakoli et al., 2022).

2.	In the abstract, they give changes in the IC50s of the drugs, but they do not attach the changes to a specific drug, so its hard for a reader to know which drugs had the changes.

Response: Thank you for the comment. This section has been revised as follows for easy readability. The section reads, “A significant decrease in median 50% inhibition concentrations (IC50s) and interquartile range (IQR) was noted; AQ from 2.996 ng/ml [IQR=2.604-4.747, n=51] in 2008 to 1.495 ng/ml [IQR=0.7134-3.318, n=40] (P<0.001) in 2019, QN from 59.64 ng/ml [IQR=29.88-80.89, n=51] in 2008 to 18.10 ng/ml [IQR=11.81-26.92, n=42] (P<0.001) in 2019, CQ from 35.19 ng/ml [IQR=16.99-71.20, n=30] in 2008 to 6.699 ng/ml [IQR=4.976-9.875, n=37] (P<0.001) in 2019 and ART from 2.680 ng/ml [IQR=1.608-4.857, n=57] in 2008 to 2.105 ng/ml [IQR=1.266-3.267, n=47] (P=0.0012) in 2019, implying increasing parasite sensitivity to the drugs over time. Abstract section, Page 1, line 58-63.”

3.	In the statistical analysis, they mention, they used chi-square test to test for the trends, I think the authors used the chi-square test for trends also known as the Cochran-Armitage trend test. This should be very clear in the methods, because for most analyses the typical chi-square test could not be used in most of those analyses.

Response: Thank you for your observation. The chi-square test for trends is also called the Cochran –Armitage test for trends. Chi-square/ Cochran Armitage test for trends was used to establish the frequencies and trends of polymorphisms at individual codons in the methods under statistical analyses section, Page 10, Line 302.

4.	I think the authors ignored a very interesting finding. The lumefantrine IC50s increased to 19.38ng/ml in 2018-2019 from 11.17 in 2013-2014 and 2008-2009. Even though this trend p-value was not significant, there was almost a 2 fold increase in the IC50s. In my opinion, this should be discussed as something of concern, but stating that this was not statically significant, given the fact that most upcoming literature is showing the same trend especially in areas where AL is being used.

Response. Concur. Thank you for the comment.This has been included un the manuscriptin line 436 to 440. The section reads, “). LU median IC50S increased by almost two folds from 11.17 (8.273-18.740) ng/ml in 2013-2014 time point to 19.38 (3.270-33.680) ng/ml in 2018-2019 time point, although this was not statistically significant (P=0.2692).”

5.	In the discussion, the authors describe why they chose the 3 time points, in my opinion this should be in the methods giving a rationale for picking these 3 time points, other than going to the discussion section

Response: Thank you for your observation. This is a great suggestion. The description has been moved to the methods section under statistical analyses Page 10-11, line 304-316 and removed from the discussion section.

Reviewer #2: Summary: Winnie Okore et al intended to identify if there are mutation (SNPs) in Plasmodium falciparum and if these mutations confer resistance to common antimalaria in use among Kenya populations. They also set out to determine if malaria parasites are still sensitive to these anti-malarial agents. They used samples from a primary longitudinal study with objective of monitoring drug resistance and malaria epidemiology among Kenyan populations.

Comments

1.	Title does not depict the key message to the public health perspective. Something like “Increased sensitivity of malaria parasite strains to common antimalarial drugs in use by Kenyan populations” would be more captivating and appealing to policy makers, public health gurus, pharmaceuticals, and other non-biomedical scientists. Dr. Akala/LTC Kamau

Response: concur that the current title is silent on the implication. The reason is that clinical results from tiral need to accompany the report for us to go for the proposed title. If we go for it now, reviewers will return to that it is an overtone, on the account of the threshold of non in vivo work

2.	Abstract

Grammatical and composition issues

…have previously been reported to confer resistance to Artemisinin and its partner drugs in Southeast Asia…what do you mean by “its partner drug”…In my opinion, I would put it like…”have previously been reported to confer resistance to Artemisinin based Combination Therapies (ACTs).

Response: Thank you for the comment. This is a great suggestion. The statement by its partner drug has been replaced with “Artemisinin based Combination Therapies (ACTs). Abstract section, page 1, line 52.

a.	I am not comfortable with the statement..”assayed for SNPs”….assayed should be used when your test methods give quantitative details, but in your case, you simply detected presence or absence of the SNPs…would propose something like…tested for the presence of SNPs.

Response: Thank you for the comment. This is a great suggestion. The statement “assayed for SNPs “has been replaced with “tested for the presence of SNPs. Abstract section, page 1, line 53-54.

b.	The sentence starting with.. “Nonetheless” can be replaced by “However”…There is mismatch in statement “mutation at 876 were not significantly associated with” .. I suggest either “mutation at 876 did not significantly affect the performance of selected antimalarial at IC50S” or “there was no statistical significance between mutation at 876 and parasite sensitivity to selected antimalarial.

Response: Thank you for suggestion. The word “Nonetheless” has been replaced with “However”. Also, the statement “mutation at 876 were not significantly associated with” has been replaced with “there was no statistical significance between mutation at 876 and parasite sensitivity to selected antimalarials.” Abstract section, page 1, line 63.

c.	The statement “Kenyan parasites are sensitive” is ambiguous … The parasites are not made in Kenya or restricted to Kenyan population. A statement like “malaria parasites among Kenyan populations are still sensitive to…. Despite the presence of Pfmrp1 in parasites among the population. ” would be more befitting.

Response: Thank you for the suggestion. The statement “Kenyan parasites are sensitive….” has been replaced with “malaria parasites among Kenyan populations to AQ, QN, CQ, ART, LU and MQ. Despite the presence of Pfmrp1 mutations in parasites among the population for clarity. Abstract section, page 1-2, line 65-79.

Technical issues: before you conclude that there is increased or even just sustained sensitivity, kindly clarify the following issues.

a.	is the composition of the active substance in those selected drugs the same for the entire study period? If yes, how did you prove/measure it?

Response: Our lab subscribes to the WorldWide Antimalarial Resistance Network (WWARN) in vitro Scientific group EQA program. For the past 15 years, this EQA program has been supplying our lab with new aliquots of drug molecules that pass their QC on annual basis to replace the previous supplies documentation related to the quality of drugs is filled in the lab.

b.	Were the drugs procured from the same Manufacturer or vendor for the entire study period?

Response: Our lab subscribes to the WorldWide Antimalarial Resistance Network (WWARN) in vitro Scientific group EQA program. For the past 15 years, this EQA program has been supplying our lab with new aliquots of drug molecules that pass their QC on annual basis to replace the previous supplies documentation related to the quality of drugs is filled in the lab

c.	There are so many confounding that can lead to that outcome, ranging from patient’s factors, parasites factors, environmental factors, therapeutics, technical competency factors among others. How did you control for these variables/factors in your study?. Without rigorously controlling for these factors, you cannot confidently conclude that there was increased sensitivity. – Dr. Akala/LTC Kamau

Response: Thank you for your comment. The are grateful you catch all the confounders. Our previous work details how we control for these confounders (Akala et al., 2011, Cheruiyot et al., 2016). For immediate ex vivo, blood sample is centrifuged to remove buffy coat plus serum. The pellet is then re-suspended in 11mL wash media to homogeneity then centrifuged. This is repeated three times to reduce hose immune factors and cells that would confer effects to the assay. However, as we move towards personalized medicine, it may appear that in the near future, we “humanize” assays as much as possible by assaying samples without controlling for these host confounders so as to accurately quantify drug action, mimicking natural settings.

3.	Introduction

a.	First paragraph is mixed up and colloquial….It should read something like; prior to COVID19 pandemic, substantial gains were made towards malaria eradication as disease burden had significantly reduced in the past decade. Unfortunately, COVID19 pandemic neutralized these gains as transmission intensity between 2020 to 2023 exceeded that of the previous years….So many sentences are hanging, words have been misplaced or misused among others. I suggest the entire introduction should be re-written in standard grammar and English composition.

Response: We are grateful for your comment. The first paragraph has been replaced with the statement suggested. Further, most of the sentences in the introduction section have been rephrases as appropriate.

4.	Materials and methods

Study sites & sample collections:

a.	The paragraph starting with..”This retrospective study…” I guess should be like “This study retrospectively analyzed samples collected between 2008 and 2019…

Response: Thank you for the suggestion. The statement “this retrospective study analyzed samples collected between 2008 and 2019” was replaced with the suggested phrase page 6, line 214.

b.	the statement “under the protocol”, Is this the name of the primary study or you actually meant the primary study which your current study was nested in?

Response: Thank you for the comment. The statement referred to the parent study, under which the current study is an objective.

On Ethical approval, you indicated that this study was approved by KEMRI REC and Walter Reed. Was this ethical approval for your study or for the primary study?,

Response: For the primary study which has the current study as an objective for inferring temporal changes.

If it was for your study, why was Walter Reed involved in the approval when the entire study was executed in Kenya?

Response: Thank you for the comment. Walter Reed Army Institute of Research (WRAIR), Institutional Review Board was involved in the approval because the primary study is funded by the U.S. Department of Defense Global Emerging Infections Surveillance and Response System (GEIS), Armed Forces Health Surveillance. GEIS is a division of the USAMRD-A which has partnered with Kenya to advance research on endemic infectious diseases and their laboratories are in KEMRI campus. The rule is that all federal funded research though a US-based institution eg. WRAIR and happening in a separate nation must receive thial approval from at least the US-based partner institution plus host-nation ERB

c.	Use more suitable joining words, adjectives and adverbs in the paragraph which start with “the samples were obtained” up to “marigat sub-county hospital” . The word “capture” may be replaced by “represent” .

Response: Concur. The word “capture” has been replaced with “represent”. Study sites and sample collection section, page 7, line 224. page 7, line 221-224.

d.	From your submission, inclusion criteria were through a positive RDT and presentation with malaria related symptoms. If yes, who screened for these symptoms and what were those symptoms, what happened to those with severe malaria. I guess these are details of the primary study which I think has been published. To save you from all these questions, cite the study and refer the readers there for details

Response: Concur. the section has been re-written to clarify that, “The clinician performed diagnosis by assessing for symptoms including; headache, vomiting, coughing, diarrhea, body temperature and body weight. Those with severe malaria were not eligible for ……” page 7, line 227 and 235.

e.	You used the abbreviation “AL”, Is it a standard abbreviation in use and what does it mean since you did not define it in the abstract?. That dosing of AL (I guess stands for Artemisinin/Lumefantrine) of 8 hours and followed by 12 hours, is that the standard in the Kenyan Clinical guideline for treatment of uncomplicated malaria, kindly be sure?

Response: Thank you for the comment. Yes, it is a standard abbreviation which stands for Artemether Lumefantrine-(AL) it has been first mentioned and defined in the introduction section page 4, line 150. We are sure artemether-lumefantrine (AL) in accordance with the Kenya Ministry of Health guideline on uncomplicated malaria treatment is administered after 8 hours day 1 and then after 12 hrs for subsequent two days (Kenya National guideline on malaria treatment, 2016).

5.	Genotypic analysis of Pfmrp1

a.	What do you mean by “genomic DNA”?, is there another type of DNA for Plasmodium falciparum?..just putting parasite DNA would be sufficient.

Response: Thank you for your observation it was an oversight error. Genomic DNA has been replaced with parasite DNA in the methods section, genotypic analysis of Pfmrp1 gene, page 8 and line 251.

b.	Put a proper negative sign where you write -20. Also degree centigrade should be oc, not oC as you put it.

Response: Thank you for your observation it was an oversight error. -20 and (oC) has been corrected to oc and -20 (minus symbol inserted) and rewritten in the methods section, genotypic analysis of Pfmrp1 gene page 8 and line 252.

Were the primers designed in house or commercially procured?.

Response: Thank you for the comment. The primers were designed in house and commercially procured afterwards from Integrated DNA Technologies, the primers used are in supporting information S1 Table file

c.	How did you perform quality assurance of matrix assisted laser desorption ionization-Time of flight mass spectrometry?,

Response: Thank you for the comment. Quality assurance was performed a follows:- First, from the iPLEX assay design, no two alleles were within 15 Daltons of each other thus greatly reducing any allele-bias. Secondly, the MALDI-TOF platform uses a 3-point calibrant (three unique oligonucleotides of known mass) in each run that establishes an equation for the best-fit curve for the data. (Ragoussis et al., 2006). Lastly, quality control reactions were also included in each run; inclusion of a DNA positive control, inclusion of a well subjected to PCR and iPLEX reaction but lacks any DNA and a well with Taq polymerase but not subjected PCR stage. All these are run in duplicates and assist in evaluating background noise.

Kindly include a section on its Quality assurance.

Response: Thank you for the comment. Section on its quality assurance has been included in the methods section, genotypic analysis of Pfmrp1 gene, page 8, line 259-264.

Are there instances on the MALTI-TOF MS platform where you can obtain pure SNPs which are all mutants as you have put in your characterization?

Response: Thank you for the comment. Yes, this particular assay combines the specificity of a single-base extension PCR (SBE) known as iPLEX PCR and the accuracy of MALDI-TOF. The calls for the targeted genotypes are clustered as either, low mass homozygous, high mass homozygous and heterozygous. In my characterization high mass homozygous and heterozygous were all characterized as mutants. methods section, genotypic analysis of Pfmrp1 gene, page 8, line 258.

6.	Drug preparation:

a.	The first paragraph is confusing. I guess you meant.. Drug/disc concentration ranging from …..to….. was serially diluted in a 96 well culture plate. What was the dilution factor and why the factor?

Response: Thank you for your observation. I concur, it has been revised for clarity. Methods section, Page 9, Line 272-279.

7.	Susceptibility testing

a.	I find so many missing links in this section which I think is the core of the lab assay. You indicated earlier that all these patients were treated with Artemisinin/lumefantrine, but then you are testing the sensitivity of the samples from these patients against six other anti-malarial?, isn’t there any antagonistic, synergistic, or additive effects between AL and these drugs in the experiment?, -

Response: Thank you for the comment. After consenting process, participant sample was collected before initiation of a 3-day treatment therapy using Artemisinin-lumefantrine (AL) in accordance with the Kenya Ministry of Health guidelines for malaria management. Samples collected prior to treatment was then transported to the laboratory and tested against the artemether alone, lumefantrine alone and other srugs alone so as to understand how the infection responds to policy recommended treatments, as well as non-policy recommended drugs. This data us useful for guiding futre decision of drugs to replace current treatment. the study did not perform any combination assays therefore cannot comment on nature of interaction of any oof the tested drugs against the infections assayed here.

b.	What is the T1/2 of AL once in the serum?.

Response: Thank you for the comment. The half-life of artemether is 2-3hrs and that of lumefantrine is 3–4 days. This was not relevant to in vitro or ex vivo assays here since they were carried out in the absence of host CYP 3A4 that would be metabolizing the drugs.

c.	After administration of A/L, how long did you wait before setting up the experiment for the other drugs?

Response: Thank you for the comment. Whole blood containing the parasite (since the participant had been confirmed to habor parasites) was drawn from a consenting individual, put in a vacutainer and kept on the workbench. The patient walked out of the laboratory section of the study premises to the clinician’s office, leaving the sample in the test tube on the bench at the lab. While in the clinician’s room, the patient was given water and medicine. The clinician observed the participant taking the medicine. The clinician provided the participant room to rest for one hour to assess retention of the drugs- not vomit- and generally assess vital signs. After the 1 hour, the participant was released from hospital with the AL treatment first dose in his blood already interacting with the parasites in the body. The sample that remained in the test tube was then sent to the lab where it was tested to assess how the parasite in the sample respond to each o the drugs, singly, in vitro/ex vivo (Out of the participate body). This is the data that is being reported here.

On day 7 when the participat reurned to the study team, diagnosis was done to tell whether the parasites that were in blood were still in there or were no longer there. Absence of parasites was attributed to presence of drug in the participants body.

d.	Did you culture only control strains or even parasites from the field?.

Response: Thank you for the comment. Both controls strains and field isolates (both archived and freshly collected isolates) were cultured before in vitro drug sensitivity testing. Notably, all freshly collected field isolates from far sites were cultured while for nearby sites only those that did not meet the ex vivo drug testing threshold (parastaemia < 1% at 2% haematocrit) were cultured.

e.	How did you ensure quality of results from SYBR Green dye and Tecan Genios?.

Response: Thank you for the comment. SYBR Green assays were done in three replicates as earlier described by Akala et al., 2011. Internal reference controls; W2 strain (resistant to chloroquine and sensitive to mefloquine) and 3D7/D6 (Sensitive to chloroquine and resistant to mefloquine) were assessed against all drugs tested in parallel for each field isolate tested. This assay was already optimized and adapted in the lab and its quality had already been determined by our previous study (Cheruiyot et al., 2017). Additionally, the Tecan Genios is maintained and calibrated using known fluorometric standards to ensure correct readouts of samples.

f.	Whilst the authors referred us to already published protocols or studies, I feel there is need to add more information to this section.

Response: Thank you for the comment. Clarification of the samples tested prior to AL treatment has been added in the drug susceptibility testing using SYBR green 1 assay section, page 9-10, line 284-291.

8.	Statistical analysis:

a.	What basis did you use to categorize your isolates into those years?

Response: Thank you for the comment. Samples used were collected before ACTs deployment in Kenya and during their implementation. Since it took some time for them to be implemented due to several challenges (Amin et al., 2007). The basis of categorization was a 3-year apart difference left in between the study time-points during the study period. The 3 years differentiated the transition period (2008-2009), the mid era (2013-2014) and post-ACTs era (2018-2019) during the implementation of ACTs in Kenya.

b.	Does Mann-Whitney U test measure association or correlation?

Response: Thank you for the comment. Mann-Whitney U test does independent groups and yes, it is a test of association.

c.	Why did you choose this test over Wilcoxon signed rank test?

Response: Thank you for the comment. Mann-Whitney U test is a test of independent samples while Wilcoxon signed rank test is a test of dependent samples. Also Wilcoxon signed test is for paired test. Values in each row represent paired observations, in this study IC50s were obtained by testing samples at different study periods 2008 to 2019

9.	Results:

a.	Tables should be labelled below not above.

Response: Thank you for your observation however, according to PLOS ONE formatting table guidelines the table title should be labelled above only the footnotes and legends should be labelled below as it is shown on the tables in the manuscript.

b.	Could there be any reasons why the number of mutants in 1876v are higher than that of wild types for all the study points?

Response: Thank you for your observation. Despite limited study on the role Pfmrp1 plays in drug resistance, some studies have associated mutation at codons 876 and 1390 to ACT response both via in vivo and in vitro susceptibility assays. As a result of the adoption of artemether lumefantrine (AL) in Kenya as a first line antimalarial therapy from 2006, there has been increased use of AL in the population thus increased drug pressure that could be the plausible cause of increased Pfmrp1 876V genotypes.

c.	On drug sensitivity patterns, what was the reference standard for sensitivity and who set it?.

Response: Thank you for the comment. Reference standard for sensitivity was carried out by testing of reference strains with well characterized profiles for example Dd2 (Mefloquine sensitive), D6, 3D7 (chloroquine sensitive) with standard cut off of <10ng/ml and < 45ng/ml in field isolates while W2 is chloroquine resistant with cut off value of > 45ng/ml. Mefloquine cut off in field isolates < 10ng/ml and in W2 clone. Quinine cut off is <275ng/ml. These isolates were maintained in culture with the individuals working in the culture laboratory and the thresholds were based on WWARN requirements.

d.	For table 3, add a column of confidence intervals.

Response: Thank you for the suggestion, 95% upper and lower confidence interval values have been added in table 4 created, since the values couldn’t all fit perfectly in table 3. Has been shown on Page 17.

e.	The result has not been appropriately communicated despite the enormous experiments. Dr.Akala/LTC Kamau

10.	Discussion:

a.	What do you mean by “post ACT error”?... Are ACTs being phased out in Kenya?.

Response: Thank you for the comment. No, post-ACT era in this study means after their effective implementation among the Kenyan populations following a treatment policy change in 2006. This occurred in 2008 when the global health funding was available allowing free of charge access to AL at ministry of heath facilities

b.	What do you mean by.. “can expand the remedial life of the current and upcoming cures?”.

Response: Thank you for the comment. The statement “can expand the remedial life of the present and upcoming cures” means can extend the therapeutic life of current and future treatments. (Van Tyne et al 2013) Discussion section, Page 19, line 431.

c.	Statement “returning from Kenya to Sweden” sounds inaccurate; how about we put it like.. “Returning to Sweden from Kenya”.. the destination has to come before the origin.

Response: Thank you for the suggestion. We are sorry, this was an oversight error. The statement “returning from Kenya to Sweden” has been corrected to “returning to Sweden from Kenya.” Discussion section, Page 20, line 454.

d.	When you say “samples from a traveler”; was this one person bled multiple times or it was just a grammatical error?

Response: Thank you for your observation. We are sorry, this was an oversight grammatical error it was one person bled once. “samples” has been corrected to sample. Discussion section, Page 20, line 454.

e.	What does SEA mean as you used in the discussion? As an immunologist, I have taken it for Schizont Egress Antigen.

Response: Thank you for your observation. SEA abbreviated for Southeast Asia is first mentioned and abbreviated in introduction section, Page 3, line 95-96.

f.	Why would selection pressure affect mutation at 876V only and not other alleles or loci as you seemed to be concluding?

Response: Based on earlier findings conducted in Africa, North-east Myanmar and Thai-Myanmar border, AL has been shown to select for the CQ-sensitive parasites (Sisowath et al 2009), High frequency of 876V mutations noted correspondingly in both populations (Phompradit P, Muhamad P, Wisedpanichkij R, Chaijaroenkul W, Na-Bangchang K, 2014; Achieng et al.,2015; Gupta et al 2014) might be due the use of the current treatment regimen AL against CQ-sensitive parasites.

g.	The stated limitations are so strong that it can weaken the statistical analysis and conclusions from this study. Dr.Akala/LTC Kamau

Response: Thank you for the comment. We have reworded the study limitations.

While revising your submission, please upload your figure files to the Preflight Analysis and Conversion Engine (PACE) digital diagnostic tool, https://pacev2.apexcovantage.com/. PACE helps ensure that figures meet PLOS requirements. To use PACE, you must first register as a user. Registration is free. Then, login and navigate to the UPLOAD tab, where you will find detailed instructions on how to use the tool. If you encounter any issues or have any questions when using PACE, please email PLOS at figures@plos.org. Please note that Supporting Information files do not need this step.
